# Role of Exciton Diffusion and Lifetime in Organic Solar Cells with a Low Energy Offset

**DOI:** 10.1021/acs.jpclett.2c00791

**Published:** 2022-05-12

**Authors:** Drew B. Riley, Paul Meredith, Ardalan Armin, Oskar J. Sandberg

**Affiliations:** Sustainable Advanced Materials Programme (Sêr SAM), Department of Physics, Swansea University, Singleton Park, Swansea SA2 8PP, United Kingdom

## Abstract

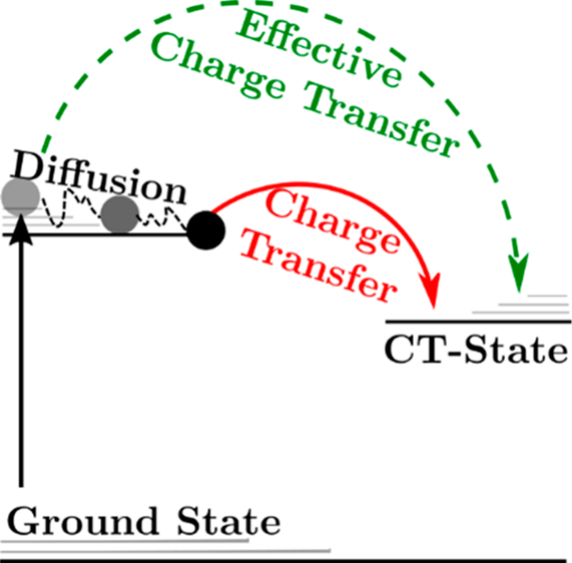

Despite general agreement that the generation of free charges in organic solar cells is driven by an energetic offset, power conversion efficiencies have been improved using low-offset blends. In this work, we explore the interconnected roles that exciton diffusion and lifetime play in the charge generation process under various energetic offsets. A detailed balance approach is used to develop an analytic framework for exciton dissociation and free-charge generation accounting for exciton diffusion to and dissociation at the donor–acceptor interface. For low-offset systems, we find the exciton lifetime to be a pivotal component in the charge generation process, as it influences both the exciton and CT state dissociation. These findings suggest that any novel low-offset material combination must have long diffusion lengths with long exciton lifetimes to achieve optimum charge generation yields.

Recently, bulk heterojunction (BHJ) organic solar cells (OSCs) made of blends of electron donating and accepting organic semiconductors have surpassed power conversion efficiencies (PCEs) of 19% with 25% being predicted.^1−4^ The recent rise in PCE has been driven by the introduction of narrow-gap nonfullerene acceptors (NFAs), which show enhanced photon absorption of the solar spectrum when used in conjunction with an appropriate and complementary electron donor. This increase in absorption combined with a superior charge generation yield (CGY) observed in state-of-the-art NFA-based BHJs ultimately cumulates in short-circuit currents (*J*_SC_) much higher than their fullerene-based predecessors.^5−8^ Concurrently, the reduction of energetic offset between donor and acceptor molecules in low-offset NFA BHJs has reduced losses associated with the open-circuit voltage (*V*_OC_).^9,10^ Specifically, low-offset NFA systems have small energetic differences between the highest occupied molecular orbital (HOMO) levels of the donor and acceptor. As such, the increase in PCE of NFAs, compared to fullerene blends, is ascribable to both the reduction of losses to the *V*_OC_, brought about by low HOMO offsets, and the increase in *J*_SC_, supported by high CGYs.

The charge generation process in OSCs is typically described stepwise from photon absorption in the active layer to free-charge carrier extraction at the device electrodes.^5,11^ Due to the low dielectric constant in organic semiconductors, the primary excitation species upon photon absorption are bound electron–hole pairs, known as excitons, which are localized to either the donor or acceptor phase.^12^ To dissociate into free-charge carriers, an exciton must first diffuse to the interface between the donor and acceptor phases of the BHJ, a process that is in competition with the radiative and nonradiative decay of the exciton. After reaching the interface, an exciton in the donor (acceptor) phase can dissociate into a charge-transfer (CT) state by transferring the electron (hole) to the acceptor (donor) phase, referred to as type I (II) charge generation.^13^ As the binding energy of CT states is much lower than that of excitons, this intermediate state can dissociate into separated free-charge states (CS states) with relatively high quantum efficiencies.^8,14^ While it is widely accepted that increasing the exciton diffusion length enhances the exciton dissociation (via more efficient transfer of excitons to the interface), the influence of exciton diffusion on the charge-transfer efficiency at the interface is not fully understood.

During exciton dissociation, the transfer of an electron (hole) from the donor (acceptor) to the acceptor (donor) phase has been historically understood to be driven by an energetic offset between the lowest unoccupied molecular orbital, or LUMO (HOMO) levels of the two materials.^5,13,15−17^ In NFA-based low-offset systems, the driving force to dissociate excitons into CT states at the interface is expected to be small due to the reduced energetic offset. Nonetheless, this has not resulted in a reduction in the CGY, but instead, near unity CGYs have been observed in state-of-the-art low-offset systems.^8^ Contributing to the understanding of how NFA BHJs can achieve high CGY in the absence of a significant HOMO offset is the motivation behind this work and a necessary step to further progressing OSC PCEs past 20%.

While a decreasing HOMO offset is expected to reduce energetic losses to the *V*_OC_, recent studies have also shown reduced nonradiative voltage losses in low-offset NFA solar cells attributable to an equilibrium between excitons localized to the acceptor phase and CT states.^17,18^ On the other hand, it has been suggested that the decreasing driving force for exciton dissociation, brought about through decreasing the HOMO offset, can be compensated for with increasing exciton lifetime.^10,19^ Further, it has been shown that photovoltaic parameters such as CGY, *J*_SC_, *V*_OC_, and PCE are contingent on not only the relative energetics of the donor and acceptor molecules but also the kinetic rate constants between excitons, CT states, and CS states. This includes the interplay between the lifetimes of excitons and CT states as well as the degree of equilibrium between the two states.^3^ However, these analyses do not consider the diffusion of excitons to the donor–acceptor interface. Instead, they assume that each exciton generated in the bulk dissociates into a CT state via charge transfer at a rate independent of exciton diffusion.

In this work, the role of exciton diffusion in exciton dissociation and charge generation yield of low-offset organic solar cells is investigated. An expression for the exciton dissociation efficiency and effective dissociation rate constant is derived accounting for the exciton diffusion to and dissociation at the interface. Using this expression, it is clarified under what conditions a system with low driving force for CT state formation can achieve high charge generation yield. It is found that, in low-offset systems, large exciton lifetimes serve a twofold purpose: to increase diffusion to the interface and to reduce the rate of back-transfer of CT states to excitons. Therefore, large diffusion lengths supported by long exciton lifetimes are required for efficient charge generation in low-offset systems.

The process of singlet exciton dissociation is mediated by two steps: (i) diffusion of excitons to the donor–acceptor interface and (ii) charge transfer at the interface with rate constant *k*_CT,0_. In the case of excitons in the donor (acceptor) phase, *k*_CT,0_ is the electron (hole) transfer rate constant associated with type I (II) charge generation. In the limit where diffusion to the interface is efficient the exciton dissociation rate is independent of diffusion. In this limit, the efficiency of exciton dissociation (*P*_S_) is given by the charge-transfer efficiency η_CT_ of excitons at the interface, described by the competition between the charge-transfer rate at the interface and the lifetime of the singlet exciton (τ) within the limiting phase as
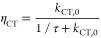
1

In general, away from the diffusion independent limit, the effective rate of exciton dissociation will be given by processes (i) and (ii) occurring in series. To derive an expression for the exciton dissociation efficiency and effective dissociation rate constant, accounting for exciton diffusion to and charge transfer at the interface, we consider a domain of length *L*, spanning 0 < *x* < *L*, in which excitons are uniformly generated at rate *G*. Figure 1 shows a schematic state diagram and the relevant rates of diffusion, decay, charge transfer, and CT state-to-exciton back-transfer for a singlet exciton in either the donor or acceptor phase. Under these conditions, the diffusion equation for excitons in the bulk takes the form
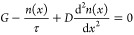
2where *n*(*x*) is the exciton density at position *x* in the domain and *D* is the diffusion coefficient for the singlet excitons. To account for exciton-to-CT state dissociation and CT state-to-exciton back-transfer, the exciton current leaving the domain at the interfaces can be expressed as
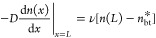
3
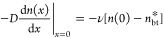
4with ν being the interfacial velocity of charge transfer from one phase to the other and *νn*_bt_^*^ being the exciton current entering the domain via back-transfer from CT states. Here, *n*_bt_^*^ is an effective density that depends on the prevailing density of CT states at the interface but is independent of *x*. The solution to eqs 2–4 is obtained as
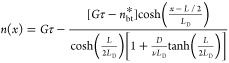
5where  is the one-dimensional exciton diffusion length.

**Figure 1:**
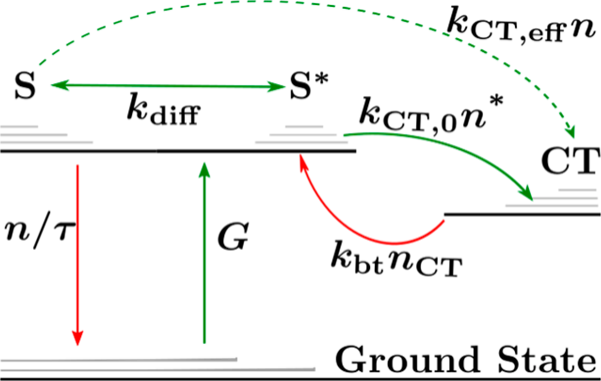
Schematic energy-level diagram showing relevant kinetics and processes occurring for bulk (S) and interfacial (S*) excitons and charge-transfer states (CT). Here, *k*_diff_ represents the rate constant of diffusion from bulk to interfacial excitonic states, *k*_CT,0_ is the electron or hole transfer rate constant from interfacial excitons-to-CT states, *k*_bt_*n*_CT_ is the rate of back-transfer from CT states to excitons, τ is the exciton lifetime, *G* is the rate of exciton generation, and *k*_CT,eff_ is the effective dissociation rate constant for all excitons.

However, eq 2 does not explicitly contain the exciton-to-CT state charge transfer and CT state-to-exciton back-transfer rates, while eq 5 strongly depends on the position within the domain. Therefore, to obtain a general rate equation that relates excitons in the bulk to excitons at the interface, one can average eq 2 across the domain to obtain
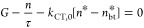
6where *n* = (1/*L*)∫_0_^*L*^*n*(*x*)d*x* represents the average density of bulk excitons, n* is the density of excitons at the interfaces, and *k*_CT,0_ = 2ν/*L*. Evident in eq 6 is that the CT state-to-exciton back-transfer rate can be equivalently expressed as *k*_CT,0_*n*_bt_^*^ or *k*_bt_*n*_CT_ (as shown in Figure 1, where *n*_CT_ is the density of CT states and *k*_bt_ is the associated back-transfer rate constant). At thermal equilibrium, the exciton-to-CT state and CT state-to-exciton rates must balance, *k*_bt_*n*_CT,eq_ = *k*_CT,0_*n*_eq_^*^, leading to
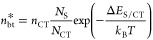
7where *k*_B_ is the Boltzmann constant, *T* is the temperature, *E*_S_ (*E*_CT_) and *N*_S_ (*N*_CT_) are the energy and available density of states for the lowest singlet exciton (CT) states, respectively, while , , and Δ*E*_S/CT_ = *E*_S_ – *E*_CT_ is the energetic offset between the exciton and CT states. Additionally, at thermal equilibrium, the net diffusion current of excitons must vanish such that *n* = n* = *n*_bt_^*^, in accordance with detailed balance.

After accounting for this, eq 6 can be equivalently expressed in terms of *n* as
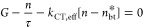
8where
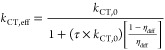
9is the effective charge-transfer rate constant for excitons generated within the domain, while η_diff_ is the efficiency of exciton diffusion to the interface given by
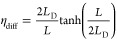
10

Finally, the overall exciton dissociation efficiency, defined as the number of dissociated excitons relative to the total number of generated excitons can be found as
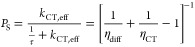
11where eqs 1 and 9 were used in the last step. Note that *P*_S_ is not given by the simple product of the diffusion and dissociation efficiencies, indicating that processes (i) and (ii) are not independent.

To substantiate eqs 9 and 11, a 1D Monte Carlo hopping model was implemented to simulate the exciton kinetics including diffusion, decay, and interfacial charge transfer. Monte Carlo simulations were used, as they have been shown to accurately account for exciton dynamics within organic semiconductors and BHJs.^20−26^ Furthermore, the use of Monte Carlo simulations allows for the calculation of the exciton dissociation efficiency and effective dissociation rate constant under conditions where the domain size, dissociation rate constant at the interface, exciton lifetime, and exciton diffusion coefficient are known precisely. The simulated dissociation efficiency and effective dissociation rate constant can then be compared to eqs 9 and 11. The details of the simulation are outlined in the Methods section. The exciton dissociation efficiency was calculated as the ratio of excitons exiting the domain at the interfaces to the total number of excitons generated in the simulation, from which the effective dissociation rate constant can be calculated through eq 11. In this formalism, the characteristic length ratio (defined as 2*L*_D_/*L*) and the lifetime-product (defined as τ × *k*_CT,0_) can be controlled by specifying the domain size and interfacial charge-transfer rate constant, respectively, while leaving the exciton lifetime and diffusion coefficient unaffected. It is important to note that, in general, these two metrics are not independent, as increases in the exciton lifetime will affect both the lifetime-product and the diffusion length. The effect on these metrics of changing the diffusion constant and lifetime are discussed throughout the remainder of this contribution. In these simulations, the exciton lifetime was 300 ps, while the diffusion coefficient was of 5 × 10^–3^ cm^2^/s, leading to *L*_D_ = 12 nm.

Figure 2 shows the normalized effective dissociation rate constant and exciton dissociation efficiency as a function of the lifetime-product for selected diffusion efficiencies, determined by the characteristic length ratio through eq 10 (Figure 2a,c), and the characteristic length ratio for various charge-transfer efficiencies, determined by the lifetime-product through eq 1 (Figure 2b,d). The circles indicate values from the Monte Carlo simulations, while the colored lines indicate eqs 9 and 11 plotted with the associated values of τ, *k*_CT,0_, *L*, and *D*. The analytic solution provided by eqs 9 and 11 reproduces the simulated *P*_S_ and *k*_CT,eff_ over the range of parameters used. As noted above, exciton dissociation can be limited by two distinct processes: diffusion to the interface and dissociation at the interface. As will be shown below, both these processes must be efficient for excitons generated in the domain to be dissociated efficiently.

**Figure 2:**
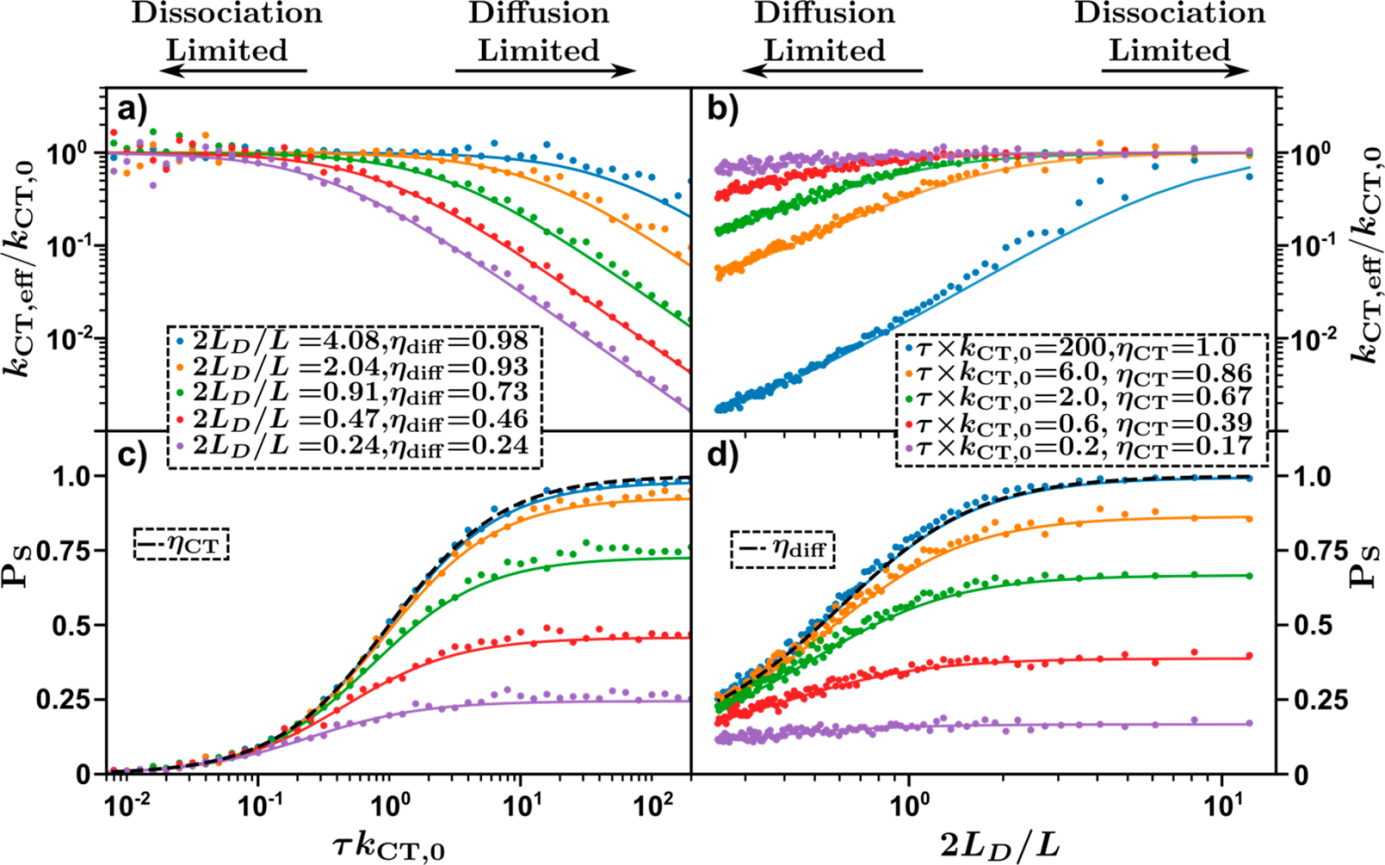
(Top) Normalized effective exciton dissociation rate constant and (Bottom) exciton dissociation efficiency as a function of (left) lifetime-product and (right) the characteristic length ratio. Circles indicate Monte Carlo simulations, solid lines indicate eqs 9 and 11, black dashed lines indicate eqs 1 and 10.

Under conditions when either τ × *k*_CT,0_ is small or 2*L*_D_/*L* is large, corresponding to η_CT_ ≪ η_diff_ or η_diff_ → 1, respectively, the overall dissociation rate of excitons is dissociation limited. As indicated on the left-hand side of Figure 2a and right-hand side of Figure 2b, this limit is characterized by *k*_CT,eff_ → *k*_CT,0_. Under these conditions, the dissociation at the interface is the rate-limiting process, and according to eq 11, *P*_S_ = η_CT_ (indicated by the black dashed line in Figure 2c). In the dissociation limited regime, *P*_S_ is strongly dependent on lifetime-product, asymptotically approaching *P*_S_ = 0 with decreasing lifetime-product. Conversely, under conditions when τ × *k*_CT,0_ is large (right-hand side of Figure 2a) or 2*L*_D_/*L* is small (left-hand side of Figure 2b), corresponding to η_CT_ → 1 or η_diff_ ≪ η_CT_, respectively, the exciton dissociation is diffusion limited. In this limit, diffusion to the interface is the rate-limiting process, resulting in *k*_CT,eff_/*k*_CT,0_ ≪ 1 and *P*_S_ = η_diff_ (indicated by the black dashed line in Figure 2d). In this limit, *P*_S_ is dependent only on the characteristic length ratio and approaches *P*_S_ = 0 for a diminishing characteristic length ratio, as excitons are unable to diffuse to the interface. Finally, under conditions when both τ × *k*_CT,0_ and 2*L*_D_/*L* exceed unity, shown on the right-hand sides of Figure 2c as η_diff_ → 1 and Figure 2d as η_CT_ → 1, eventually *P*_S_ → 1.

This analysis is summarized in Figure 3, which shows the simulated *P*_S_ (Figure 3a) and *k*_CT,eff_ (Figure 3b) as a function of the lifetime-product and the characteristic length ratio. Indicated on Figure 3 is the diffusion and dissociation limits. For a dissociation limited system, changes to the characteristic length ratio will not significantly affect *k*_CT,eff_ or *P*_S_. This can be recognized by moving vertically in Figure 3 in the dissociation limited regime. Similarly, for a diffusion limited system, changes to the lifetime-product will not significantly alter *k*_CT,eff_ or *P*_S_. This can be recognized by moving horizontally in Figure 3 in the diffusion limited regime.

**Figure 3:**
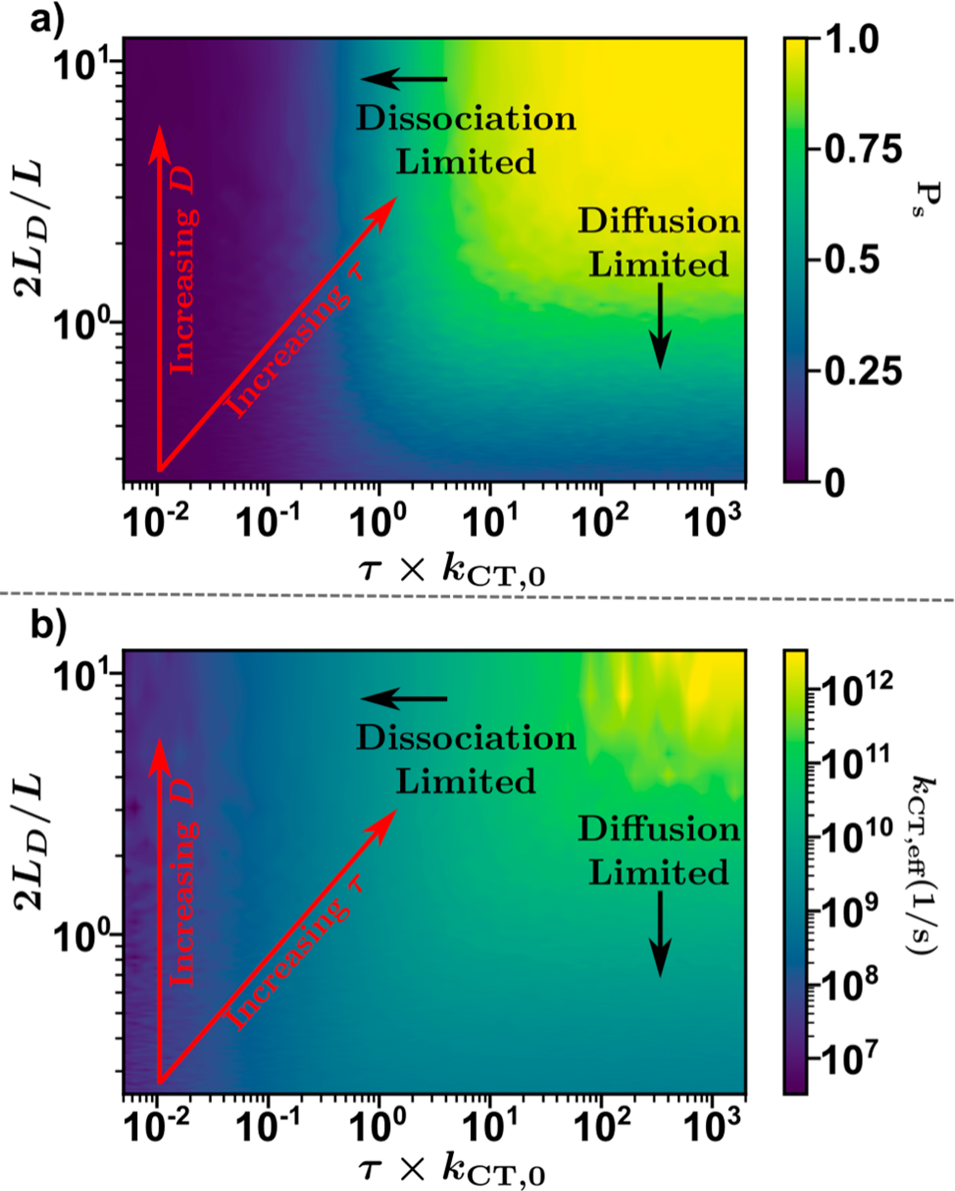
(a) Exciton dissociation efficiency and (b) effective exciton dissociation rate constant as a function of characteristic length ratio and lifetime-product. Red lines indicate the direction a system will move for an increasing exciton diffusion constant and lifetime.

These observations highlight the primary thesis of this work: to efficiently dissociate excitons into CT states, the phase limiting charge generation must simultaneously have efficient diffusion to and dissociation at the interface, enabled by high lifetime-products and characteristic length ratios. The former can be increased by increasing the exciton lifetime or increasing the dissociation rate constant at the interface. The latter can be increased by increasing the diffusion length, via increases in exciton lifetime or diffusion constant, or by decreasing the domain size.^27,28^ Therefore, the exciton lifetime plays a crucial role in determining the exciton dissociation efficiency, as increases in exciton lifetime increase both the characteristic length ratio and lifetime-product. This observation agrees with previous experimental studies by other researchers.^10^ Despite this, the diffusion constant plays an equally important role in determining the characteristic length ratio and therefore is important in determining the diffusion efficiency, the dissociation efficiency, and effective dissociation rate constant. This is highlighted by the red lines in Figure 3. Increases in diffusion constant lead to increases in the characteristic length ratio, manifesting in a vertical transition in Figure 3, while increases in the exciton lifetime increase both the characteristic length ratio as well as the lifetime-product, signified by the sloped red lines in Figure 3.

To explore the effects that exciton diffusion has on the device performance of organic solar cells, the charge generation yield (*P*_CGY_) was calculated. Here, *P*_CGY_ is defined as the ratio of generated CS states to the total number of generated excitons. To calculate *P*_CGY_, the kinetic interplay between excitons, CT states, and CS states is considered,^3^ as described in detail in the Methods section. After accounting for the generation, recombination, reformation, and dissociation of CT states and excitons, summarized in Figure 4a, we find *P*_CGY_ = *P*_S_*P*_CT_, where *P*_CT_ = *k*_d_/(*k*_d_ + *k*_f_ + *k*_bt_^′^) denotes the CT state-to-CS state dissociation efficiency. Further, *k*_d_ is the CT state dissociation rate constant, *k*_f_ is the CT state recombination rate constant, and *k*_bt_^′^ = (1 – *P*_S_)*k*_bt,eff_. Finally, *k*_bt,eff_ is the effective CT-to-exciton back-transfer rate constant related to *k*_CT,eff_ via *k*_bt,eff_ = (*n*_bt_^*^/*n*_CT_)*k*_CT,eff_; hence,
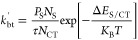
12in accordance with eqs 7 and 11. Consequentially, CT states generated directly from the ground state or via interfacial charge transfer may form excitons via this back-transfer mechanism and reform CT states many times over.

**Figure 4:**
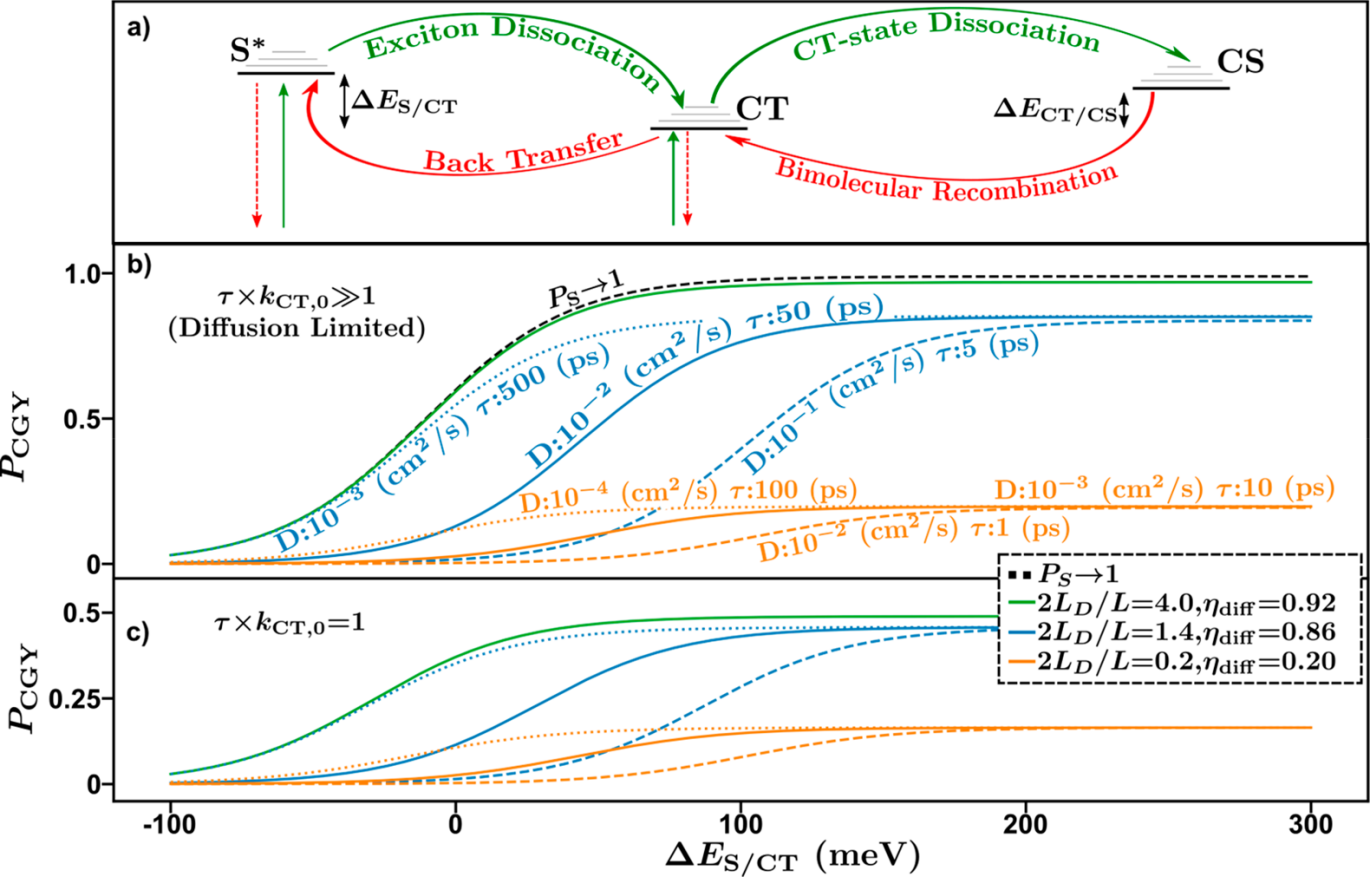
(a) Schematic energy-level diagram summarizing the work of Sandberg et al.^3^ Labeled are interfacial excitonic states (S*), charge-transfer states (CT), and charge separated states (CS), as well as the varying pathways between them and to the ground state (vertical arrows). Charge generation yield as a function of exciton-to-CT state offset for a system with (a) large and (b) unity lifetime-products. The black dashed line indicates the conclusion of Sandberg et al.^3^ Colors of lines indicate the characteristic length ratios. Moving from dotted to solid to dashed lines indicates increasing exciton diffusion constant and decreasing exciton lifetime.

The idealized case for *P*_CGY_ with efficient diffusion, corresponding to η_diff_ = 1, is shown for a high interfacial charge-transfer rate in the dashed black line in Figure 4b. The calculated *P*_CGY_ as a function of the exciton-to-CT state energetic offset (Δ*E*_S/CT_) is summarized in Figure 4b,c for the lifetime-products τ × *k*_CT,0_ ≫ 1 and τ × *k*_CT,0_ = 1, respectively, assuming a domain size of 10 nm. In the case of a high lifetime-product (equivalent to the diffusion limited regime), eq 11 simplifies to *P*_S_ = η_diff_. Therefore, as the characteristic length ratio is increased, exciton dissociation is increased. This results in an increase in *P*_CGY_ with an increasing characteristic length ratio independent of Δ*E*_S/CT_, generally seen by comparing the different colored lines in Figure 4b.

Interestingly, in the high-offset limit (Δ*E*_S/CT_ > 200 meV) *P*_CGY_ is dependent on the characteristic length ratio yet agnostic to the individual values of τ and *D*. This effect can be observed by comparing lines of the same color in the high-offset region of Figure 4b,c. On the other hand, for low-offset systems (Δ*E*_S/CT_ < 200 meV), the CT state-to-exciton back-transfer plays a more central role in determining the *P*_CGY_. In this case, the rate of CT states undergoing back-transfer to form excitons is much higher, which in turn increases the likelihood that excitons will reform and decay. Therefore, it is expected that a higher exciton lifetime will decrease the number of CT states that recombine to the ground state via an excitonic state by the back-transfer mechanism and, in turn, increase the *P*_CGY_. This effect is evident in comparing the dotted (longest τ), solid (middle τ), and dashed (shortest τ) lines of the same color (equivalent *L*_D_) in the low-offset region of Figure 4b. In this region, *P*_CGY_ for identical characteristic length ratios is increased with increasing exciton lifetime due to decreasing CT state-to-exciton back-transfer.

This highlights the secondary thesis of this work; in low-offset systems, high exciton lifetimes increase not only the exciton dissociation through increased diffusion lengths, as shown by other researchers in previous experimental studies,^10,19^ but additionally increase the CGY by ultimately decreasing the rate of CT state-to-exciton back-transfer. The inverse relationship between the CT state-to-exciton back-transfer rate and the exciton lifetime is expressed explicitly in eq 12. Interestingly, NFAs show a significant increase in diffusion length compared to their fullerene predecessors, which would help to explain the improvements in CGY. However, it has been shown that this increase is due primarily to increases in diffusion constants.^26,29−31^ The results from Figure 4b suggest that CGY in NFA blends could be dramatically increased by focusing on blends containing low-offset acceptors that have long diffusion lengths supported by increased exciton lifetimes. Systems of this type would increase the diffusion of excitons to the interface while simultaneously reducing CT state-to-exciton back-transfer losses.

Figure 4c shows the equivalent analysis for systems with the same characteristic length ratios but with a lifetime-product of unity, corresponding to η_CT_ = 0.5. Under these conditions, the exciton dissociation efficiency is expressed as *P*_S_ = η_diff_/(1 + η_diff_), resulting in a maximal efficiency of 0.5. In general, decreases in the exciton lifetime will decrease the exciton dissociation efficiency via decreases to both the characteristic length ratio, as described above, and the lifetime-product. This additional effect is equivalent to excitons reaching the interface but being unable to dissociate into CT states, effectively “reflecting” off the interface. Therefore, even in the case of high characteristic length ratios and high energetic offsets, such as that shown by the green curve in Figure 4c, the *P*_CGY_ is reduced via reductions to the effective exciton dissociation rate constant.

In conclusion, the role that exciton diffusion plays in exciton dissociation in BHJ OSCs was investigated and analytic expressions for the exciton dissociation efficiency and the effective dissociation rate constant were derived. This analysis revealed that the exciton dissociation efficiency is determined by the efficiency of exciton diffusion to the interface (determined by the characteristic length ratio, 2*L*_D_/*L*) and the charge-transfer efficiency at the interface (determined by the lifetime-product, τ × *k*_CT,0_). The expression for exciton dissociation efficiency was used to calculate the theoretical charge generation yield in BHJ OSCs. For high-offset systems, it was found that the charge generation yield is governed by the characteristic length ratio and that the individual values of exciton lifetime and diffusion coefficient were inconsequential. However, in low-offset systems, the exciton lifetime influences not only the characteristic length ratio but also the rate of back-transfer from CT states to excitons. Therefore, the exciton lifetime plays a more critical role than the diffusion constant in determining the charge generation yield in low-offset systems. This work provides a framework for discussing the effect exciton diffusion has on both the exciton dissociation and the charge generation yield in organic solar cells, from which other photovoltaic parameters can be calculated. Our analysis suggests that future materials developed for low-offset organic bulk heterojunction solar cells must exhibit high diffusion lengths to support efficient exciton dissociation and that these diffusion lengths must include long exciton lifetimes to support efficient CT state dissociation.

## Methods

*Monte Carlo Simulations*. With the aim of modeling the exciton dynamics within an organic semiconductor, a 1D Monte Carlo hopping model was invoked.^25^ First, a domain of size *L* with lattice spacing (d*x*) is created, and the lattice is randomly populated with an exciton and allowed to evolve in time with a temporal time step size (d*t*). The diffusion constant in the film is determined as *D* = d*x*^2^/2d*t*. The lattice spacing, temporal step size, and exciton lifetime (τ) were 1 nm, 1 ps, and 300 ps respectively, corresponding to a diffusion coefficient of 5 × 10^–3^ cm^2^/s and diffusion length of 12 nm (). To explore the dependence on the characteristic length ratio (2*L*_D_/*L*), the domain size was varied from 2 to 100 nm. To investigate the effect of the lifetime-product (τ × *k*_CT,0_), the interfacial velocity of charge transfer was varied. This was accomplished by considering the rate of dissociation at the interface (*k*_int_) to be related to the interfacial velocity as ν = *k*_int_d*x*/2, and therefore, *k*_int_ = *k*_CT,0_(*L*/d*x*). The exciton dissociation efficiency was calculated as the ratio of excitons exiting the domain at the interfaces to the total number of excitons generated in the simulation, from which the effective dissociation rate can be calculated through eq 11.

*Charge Generation Yield Calculations*. In accordance with eq 6 in the main text, accounting for diffusion to and dissociation at the interface, under steady-state illumination, the kinetics of excitons and CT states is governed by the rate equations
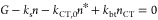
A1
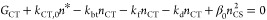
A2where *G* (*G*_CT_) is the generation of excitons (CT states) from the ground state, *n* is the spatially averaged density of excitons in the domain, n* is density of excitons at the interface, *k*_s_ is the decay rate of excitons (*k*_s_ = 1/τ), *k*_CT,0_ is the dissociation rate constant of interfacial excitons, *k*_bt_ is the back-transfer rate constant, *n*_CT_ is the density of CT states, *k*_f_ is the decay rate constant of CT states, *k*_d_ is the rate constant for the dissociation of CT states into charge separated (CS) states, *n*_CS_ is the density of charge separated states, and β_0_ is the bimolecular recombination rate constant of CS states to CT states. However, as established in Section 2 of the main text, after accounting for exciton diffusion to the interface, the net exciton-to-CT rate can be equivalently expressed as *k*_CT,0_n* – *k*_bt_*n*_CT_ = *k*_CT,eff_*n* – *k*_bt,eff_*n*_CT_, where *k*_bt,eff_ = *k*_bt_*k*_CT,eff_/*k*_CT,0_ and *k*_CT,eff_ is the effective dissociation rate constant described by eq 9. Hence, eqs A1 and A2 can be rewritten as
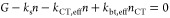
A3
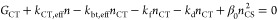
A4

Then, based on eqs A3 and A4, and following the formalism of Sandberg et al.,^3^ the CT state-to-CS state dissociation efficiency can be calculated from the decay rate of CT states, the CT state-to-CS state dissociation, and back-transfer rate from CT states to excitons as *P*_CT_ = *k*_d_/(*k*_bt_^′^ + *k*_d_ + *k*_f_), where *k*_bt_^′^ = (1 – *P*_S_)*k*_bt,eff_ is the ultimate (effective) back-transfer rate constant. Using a detailed balance approach, the back-transfer rate constant can be calculated as
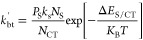
A5where *N*_S_ and *N*_CT_ are the density of states for singlet excitons and CT states, respectively, and Δ*E*_S/CT_ is the energetic difference between the exciton and CT state. The rate constant for CT state-to-CS state dissociation can be calculated as
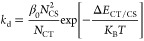
A6where *N*_CS_ is the density of states for CS states and Δ*E*_CT/CS_ is the effective CT state binding energy. The charge generation yield can then be expressed as the product of the exciton dissociation efficiency and the CT state dissociation efficiency.
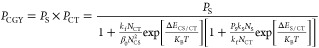
A7

The calculations in Figure 4b,c were performed by evaluating *P*_S_ from eq 11 and using the result to calculate *P*_CGY_ from eq A7. In this work, the default values for the parameters were taken from Sandberg et al. as *k*_f_ = 10^10^ s^–1^, β_0_ = 5 × 10^–10^ cm^3^ s^–1^, *N*_CT_ = 10^18^ cm^–3^, *N*_S_ = *N*_CS_ = 3 × 10^20^ cm^–3^, *T* = 300 K, and Δ*E*_CS/CT_ = 100 meV. Meanwhile, the values for *k*_S_ and *P*_S_ are determined by the choice of τ and *D*, as shown for each calculation in Figure 4b.
